# He Lives and He Reigns

**DOI:** 10.3390/jcm14228191

**Published:** 2025-11-19

**Authors:** Dimitrios E. Magouliotis, Vasiliki Androutsopoulou, Dimitrios Zacharoulis

**Affiliations:** 1 Department of Cardiac Surgery Research, Lankenau Institute for Medical Research, Main Line Health, Wynnewood, PA 19096, USA; 2 Department of Cardiothoracic Surgery, University of Thessaly, Biopolis, 41110 Larissa, Greece; androutsopoulouvasiliki@uth.gr; 3 Department of Surgery, University of Thessaly, Biopolis, 41110 Larissa, Greece; zacharoulis@uth.gr

Among the most enduring legends of the Greek seas is that of Thessalonike, the sister of Alexander the Great. After her brother’s death, she was said to have been transformed into a mermaid who wandered across the Aegean, halting ships to ask a single question: “Does King Alexander live?” The sailors’ answer determined their fate. If they replied, “He lives and reigns and rules the world,” the sea remained calm, and their voyage continued in peace; but if they said he was dead, the waves rose in fury to destroy them. Beneath this tale lies a timeless truth: it is not enough merely to live; one must live and reign [1].

This allegory captures the evolution of modern surgery and the science of quality improvement. For decades, surgical success was defined by a single, binary measure: survival. Did the patient live? Yet survival alone is only the beginning of the story. True quality demands a deeper, more demanding question, one that echoes Thessalonike’s cry across centuries and seas: Does the patient live and reign? Does the operation restore vitality, independence, and dignity? Does it return the patient not simply to life but to the sovereignty of living?

The contemporary movement for surgical quality improvement reflects this shift. Recent evidence shows that implementing patient-reported outcome measures (PROMs) at a national scale is feasible and crucial to capturing the full patient journey [2]. Surgical QI collaboratives, particularly in the UK, have not only demonstrated effectiveness but also underscored the need for improved methodology and design [3]. Frameworks for scaling surgical quality across global contexts have emerged, offering structured pathways to reduce morbidity and mortality through systems innovation [4]. Principles tailored for frontline surgical QI emphasize feasibility, resource optimization, and sustainable implementation in small-scale settings [5]. Continuous learning models in cardiothoracic surgery have shown how data transparency and shared responsibility can transform outcomes across regions [6,7,8,9].

Quality improvement in surgery thus represents far more than the refinement of processes or the pursuit of lower mortality; it is a cultural transformation—a collective reimagining of what it means to heal. Each iterative audit, protocol, and multidisciplinary discussion seeks to calm the turbulent waters that follow an operation and ensure that our patients not only survive the storm but also emerge whole, empowered, and reigning once more over their own lives.

The articles presented in this Special Issue of the *Journal of Clinical Medicine* embody this evolution. They move beyond technical success to explore how incremental refinements (e.g., in anesthetic strategy, infection control, or system design) translate into meaningful outcomes for surgical patients.

In the realm of perioperative optimization, Kim et al. demonstrate that dexmedetomidine-based opioid-sparing anesthesia in minimally invasive pectus excavatum repair significantly reduces opioid consumption and pain without compromising hemodynamic stability [10]. Similarly, Tsaousi et al. synthesize global evidence on prophylactic antiepileptic drugs after craniotomy, offering a network-meta-analytic framework that refines decision-making in neurosurgical practice [11]. Both studies remind us that perioperative care is not ancillary; instead, it is integral to recovery and quality.

At the interface of surgical craftsmanship and outcomes, Magouliotis et al. compare transthoracic clamping and endoaortic balloon occlusion in minimally invasive mitral valve surgery, revealing equivalent safety and highlighting evidence over convention as the foundation of progress [12]. In the same context, Burysz et al. expanded this philosophy to aortic interventions, demonstrating that thoracic endovascular repair can be safely performed even in medium-volume centers by experienced cardiac surgeons, affirming that quality is not the privilege of size but of structure and commitment [13].

The domain of infection control and surgical sterility is illuminated by Duffy et al., who reveal that skin-dwelling bacteria survive standard preoperative antisepsis, reminding us that even when protocols seem perfect, unseen margins of risk persist [14]. Weiss et al. and Elgabsi et al. explore procedural refinements in biliary and appendiceal surgery, demonstrating how stent dwell time, morphology, and timing influence readmissions and recurrent events [15,16].

Quality, however, transcends the operating room. Xanthopoulos et al. showed that the initiation of sodium–glucose co-transporter 2 inhibitors at discharge improved outcomes in hospitalized heart failure patients, illustrating the continuity between surgical and medical optimization [17]. Likewise, Huh and Hwang’s review on anesthetic influences in lung cancer recurrence reframes the perioperative period as a biological opportunity for improving long-term oncologic control [18].

Together, these contributions reaffirm a unifying message: quality improvement is not a static checklist but a living dialogue between science, systems, and humanity. Each dataset, each analysis, each reflection contributes a verse to the same enduring question—Does the patient live and reign?

As editors, clinicians, and scientists, we are entrusted with ensuring that our collective answer, grounded in data and empathy, remains steadfast:

Yes—he lives, and he reigns.

## Data Availability

The data that support the findings of this study are available from the corresponding author, upon reasonable request.
